# Role of Protein Kinase A Activation in the Immune System with an Emphasis on Lipopolysaccharide-Responsive and Beige-like Anchor Protein in B Cells

**DOI:** 10.3390/ijms24043098

**Published:** 2023-02-04

**Authors:** Daniela Pérez-Pérez, Leopoldo Santos-Argumedo, Juan Carlos Rodríguez-Alba, Gabriela López-Herrera

**Affiliations:** 1 Immunodeficiencies Laboratory, National Institute of Pediatrics (INP), Mexico City 04530, Mexico; 2 Doctorate Program in Biological Sciences, Autonomous National University of Mexico (UNAM), Mexico City 04510, Mexico; 3 Biomedicine Department, Center for Research and Advanced Studies of the National Polytechnic Institute (CINVESTAV), Mexico City 07360, Mexico; 4 Flow Cytometry Unit, Health Sciences Institute, Veracruzan University, Veracruz 91090, Mexico; 5 Department of Immunology, Superior School of Medicine, National Polytechnic Institute (IPN), Mexico City 11340, Mexico

**Keywords:** PKA, AKAPs, immune dysregulation, cellular mechanism, antigen receptor signaling

## Abstract

Cyclic AMP-dependent protein kinase A (PKA) is a ubiquitous enzymatic complex that is involved in a broad spectrum of intracellular receptor signaling. The activity of PKA depends on A-kinase anchoring proteins (AKAPs) that attach to PKAs close to their substrates to control signaling. Although the relevance of PKA-AKAP signaling in the immune system is evident in T cells, its relevance in B and other immune cells remains relatively unclear. In the last decade, lipopolysaccharide-responsive and beige-like anchor protein (LRBA) has emerged as an AKAP that is ubiquitously expressed in B and T cells, specifically after activation. A deficiency of LRBA leads to immune dysregulation and immunodeficiency. The cellular mechanisms regulated by LRBA have not yet been investigated. Therefore, this review summarizes the functions of PKA in immunity and provides the most recent information regarding LRBA deficiency to deepen our understanding of immune regulation and immunological diseases.

## 1. Introduction

Protein kinase A (PKA) comprises a family of serine-threonine kinases and depends on cyclic adenosine monophosphate (cAMP) for its activity. This enzyme has two regulatory subunits that dissociate upon binding to cAMP, thus allowing its catalytic subunits to reach their activation targets [1]. One mechanism that maintains the balance between the active and inactive states of PKA is the binding of A-kinase anchoring protein (AKAP) to regulatory subunits and other molecules susceptible to PKA activation, thereby bringing PKA into close proximity with its substrates. The AKAPs are classified according to their specificity for PKA regulatory subunits [2,3]. These proteins also have domains that interact with other molecules susceptible to PKA activation [2,3].

PKA is involved in embryonic development and metabolism, and the distribution of PKA isoforms differs among epithelial cells, cardiac cells, neurons, and immune cells. This review highlights the current understanding of the functions of cAMP and PKA in immune cells, specifically B lymphocytes. The presence of PKA in T cells suggests that PKA is essential for the inhibition of cell activation and proliferation. A specific PKA isoform in common variable immunodeficiency (CVID) and systemic lupus erythematosus (SLE) is associated with increased T lymphocyte activation and diminished IL-10 production [4,5,6,7,8].

However, the functions of PKA in humoral responses remain unclear. Thus, we reviewed the current knowledge regarding the functions of PKA and AKAPs in B lymphocytes. We also reviewed CVID, as it can be associated with AKAP deficiency. This disease is an inborn error of immunity characterized by hypogammaglobulinemia, recurrent bacterial infections, poor responses to vaccines, and autoimmune disorders [9]. The AKAP that is involved in CVID is lipopolysaccharide-responsive beige-like anchor protein (LRBA), which interacts with PKA in B cells [10,11].

## 2. Protein Kinase A

### 2.1. PKA Signaling

Protein kinases are a broadly distributed group of enzymes that catalyze gamma-phosphate transfer from adenosine triphosphate (ATP) to an amino acid in the presence of two magnesium ions (Mg^2+^) [12]. These enzymes are classified depending on whether serine, threonine, or tyrosine is phosphorylated and they regulate numerous signaling pathways involved in apoptosis, cellular growth, activation, differentiation, and proliferation [13]. Protein kinase A represents a family of cytoplasmic serine/threonine kinases with various isoforms [14] and structurally comprises two regulatory and two catalytic subunits.

Activation of PKA depends on elevated intracellular cAMP levels as a result of signaling initiated by the activation of G-protein-coupled receptors (GPCRs). These receptors sense specific extracellular signals as hormones or chemokines and transduce them downstream [15]. The activation of GPCRs triggers a conformational change and confers guanine nucleotide exchange factor activity. During the activation of heterotrimeric G-proteins, the Gα subunit dissociates from the Gβ and Gγ subunits [16]. The Gα isoforms (Gαs and Gαi) are involved in signaling pathways that stimulate (Gαs) or inhibit (Gαi) the activity of adenylyl cyclases (ACs), which catalyze the formation of cAMP via ATP [17]. After the cAMP concentration is increased and signals are propagated, cAMP levels are regulated by phosphodiesterases that convert cAMP into 5’AMP. This reduces intracellular cAMP concentrations following PKA inactivation [18]. The exchange proteins directly activated by cAMP (Epac1 and Epac2) are also components of cAMP signaling that are implicated in processes such as cell adhesion. 

The activity of cAMP regulates various cellular processes such as cellular growth, proliferation, differentiation, and apoptosis. Some regulatory mechanisms ensure that PKA signaling is controlled, hence preventing the development of disorders such as cancer, autoimmunity, or immunodeficiency. Notably, the structure of components in the cAMP signaling pathway plays a crucial role.

### 2.2. Structure of PKA 

The molecular weight (MW) of the PKA holoenzyme is ~250 kDa, varying depending on the isoform and the organism, and it is expressed in the cytoplasm and nucleus. In the inactive state, PKA forms a tetrameric holoenzyme that is usually bound to AKAPs [1]. Increased cytoplasmic cAMP levels cause the holoenzme to interact with PKA regulatory subunits. This results in dissociation of the two PKA catalytic subunits that phosphorylate specific serine and/or threonine residues in R-R-X-S/T motifs (where R is arginine, X is any amino acid, S is serine, and T is threonine residues) in PKA targets [19,20].

### 2.3. PKA Catalytic Subunits

The catalytic PKA subunits in humans have a molecular weight of 43 kDa and are structurally composed of two parts; one part is a small fragment associated with ATP binding and is located near the amino terminus, and the other part is a docking site for protein substrates and is located at the carboxyl terminal, where phosphate is transferred [21]. Two catalytic subunits, Cα and Cβ, in the other part are encoded by *PRKACA* and *PRKACB*, respectively. The Cα isoforms, Cα1 and Cα2, are expreseed ubiquitously and sperm-specifically, respectively. Cβ, encoded by *PRKACB*, has four isoforms: Cβ1, Cβ2, Cβ3, and Cβ4. The expression of Cβ1 is ubiquitous, whereas Cβ2 and Cβ3 are specifically expressed in immune and neuronal cells, respectively. This tissue-specific distribution is attributed to different functions of the subunits because their activities depend on cell type, cell differentiation state, activation status, and epigenetic factors [22]. 

Moreover, Cα1 and Cβ2 do not fully dissociate from regulatory (R) subunits after adding excess cAMP. Complete dissociation is achieved only by adding a PKA substrate [23]. This suggests that PKA requires binding to protein targets for activation and explains the importance of enzyme and substrate proximity [20]. 

The holoenzyme binds to an AKAP during the canonical activation of catalytic subunits, and cAMP binding to the regulatory subunits releases the catalytic subunit. However, the non-canonical activation of PKA involves possible associations between the catalytic subunit and the components of other signaling pathways, such as nuclear factor κ-light-chain-enhancer of activated B cells (NF-κB). The NF-κB family of transcription factors is essential for the gene expression of inflammatory and immune mediators. The Cα1 isoform can interact with the protein inhibitor of κB (IκB) in the inactivated state of NF-κB1 (formed by the p50/p65 dimer). Activation of NF-κB1 results in the release of IκB, and the interaction with Cα1 leads to cAMP-independent activation of the catalytic subunit of PKA [20]. This activation mechanism is important because PKA can phosphorylate the p65 and p50 components of NF-κB that are essential for DNA binding and transcriptional activity [24]. 

### 2.4. Protein Kinase A Regulatory Subunits

Cyclic AMP is a small molecule that is produced intracellularly from ATP and amplifies membrane receptor signals. Cyclic AMP binding to regulatory subunits induces a conformational change that initiates PKA activation after the catalytic subunits are released to phosphorylate their targets [1]. The regulatory subunits are classified as RI (type I PKA) and RII (type II PKA), and the RIα and RIIα isoforms encoded by *PRKAR1A* and *PRKAR2A*, respectively, are ubiquitously expressed. The Riβ isoform encoded by *PRKAR1B* is expressed in the brain and spinal cord, whereas RIIβ, encoded by *PRKAR2B,* is expressed in the brain, endocrine system, fat, liver, immune cells, and reproductive tissues [25]. 

The RI and RII subunits of PKA have 43% identity, and RI is more responsive to cAMP than RII, which might represent a regulatory mechanism important for physiological functions. Although the reasons for this difference remain unknown, the enzymatic kinetics of the skeletal muscle (RI subunit) and heart (RII subunit) proteins have suggested that RII requires further phosphorylation after binding to cAMP, whereas RI dissociates from the C subunit depending on cAMP concentration [26]. Phosphorylation of the RII subunit is apparently dependent on the catalytic subunits of PKA that are released and activated before cAMP binds to RII subunits, constituting a mechanism of PKA autophosphorylation that occurs only in the RII subunit [27]. The regulatory subunits consist of different domains. The dimerization/docking (D/D) domain near the N-terminus mediates interactions involving AKAP scaffold proteins and homodimerization [28]. The following domain is the hinge region, where the inhibitory sequence (IS) binds and inhibits the catalytic subunit at a cleft in its active site [23]. Two cAMP-binding domains (CNB-A and CNB-B) at the carboxyl terminus are broadly conserved among isoforms and species, unlike the N-terminal region of the regulatory subunits [29].

## 3. Cyclic AMP and PKA Function in the Immune System

Over 250 known PKA substrates have numerous functions beyond cellular processes, including immune responses, and cAMP effects innate and adaptive immune cells. In general, cAMP is essential for regulating pro- and anti-inflammatory activities that impact cell proliferation, adhesion, differentiation, activation, and cytokine production [30].

### 3.1. Macrophages

Macrophages are involved in innate immune responses, primarily by phagocytosing microorganisms and producing cytokines. They are also important for processes such as wound healing and tissue repair after inflammatory responses. Macrophages can switch from pro-inflammatory (Ly6Chigh in mice, M1 in humans) to anti-inflammatory (Ly6Clow in mice, M2 in humans) phenotypes. Elevated intracellular cAMP levels in macrophages are likely to be a prerequisite for switching from the pro- to the anti-inflammatory phenotype, given that cAMP induces the expression of the orphan nuclear receptor Nr4a1 (also known as Nur77), a protein crucial for gene expression that is linked to inflammation [31]. Nuclear receptor subfamily 4 group A member (Nur77) promotes the anti-inflammatory macrophage phenotype in mice that affects inflammatory and phagocytic responses to microorganisms. The switch between anti- and pro-inflammatory profiles is generally exclusive. Some mycobacteria and fungi can switch between the inflammatory profiles through hijacking the cMAP by microbial adenylyl and guanylyl cyclases as well as cyclase toxins [32,33]. Additionally, the use of agents that reduce cAMP levels, such as theophyllin, on macrophages and neutrophils allows *Trypanosoma cruzi* replication [34]. Additionally, N-[2-p-bromocinnamylamino-ethyl]-5-isoquinolinesulphonamide (H89) is a specific PKA inhibitor that binds to the ATP-binding pocket in the catalytic subunit of PKA. H89 leads to defective macrophage recruitment to the pleural cavity in LPS-induced mouse models of inflammation, indicating that PKA is important for the pro-inflammatory functions of macrophages [35].

### 3.2. Neutrophils

Neutrophils are abundant innate immune cells that kill microorganisms via phagocytosis by releasing toxic granules and forming extracellular traps. Neutrophils must migrate from the bloodstream to reach damaged or infected tissues. Therefore, chemotaxis and cellular adhesion are also important. When chemotaxis occurs, cytoskeletal rearrangements involve polarized integrin activation, a process that seems to be regulated by PKA. Initially, PKA is inactivated by KT5720 specifically by binding to the ATP-binding pocket in the catalytic subunit. This leads to diminished migration due to defective integrin β2 expression that consequently affects cell adhesion. Additionally, PKA activity might be necessary for asymmetrical actin cytoskeletal polarization, because KT5720 abolishes this ability [36]. 

### 3.3. Natural Killer Cells

Natural killer (NK) cells originate from a lymphocytic lineage, and although they do not possess antigen receptors, they can kill virus-infected and tumor cells. Cellular processes are destroyed by signaling through activating and inhibitory receptors that induce the release of perforins and granzymes in infected or tumor cells. NK cells can also induce apoptosis, which is mediated by the CD95 ligand (CD95L) expressed in their membranes, as binding to CD95 induces the recruitment of pro-apoptotic factors such as caspase-8. Elevated cAMP levels in NK cells can inhibit cytolytic function. Furthermore, exposing NK cells to ACs, such as dideoxyadenosine (DDA) or *cis*-*N-(2*-*phenylcyclopentyl*)-azacyclotridec-1-en-*2*-amine *hydrochloride* (MDL), blocks the destruction of K562 tumor cells. This result was reproduced using H89, a PKA inhibitor [37]. 

### 3.4. T Cells

As a component of the adaptive immune response, T cells participate in the recognition of specific foreign antigens through their receptors (TCRs). This results in the expression and release of various cytokines that influence the responses of macrophages, neutrophils, and B cells. Cyclic AMP is important for inhibiting the downstream signaling of TCRs, as this terminates the physiological effects of proliferation, cytokine production, and activation. However, this has already been reviewed in the literature [38,39]; therefore, the information below focuses on the importance of CVID.

### 3.5. B Cells

B cells are responsible for antigen presentation, suppression of immune response through IL-10 secretion, and production of specific antibodies. B cells have several developmental stages that originate in the bone, where immature B cells express a membrane immunoglobulin (IgG), following which they migrate to secondary lymphatic organs to end their maturation and are finally activated by antigen exposure [40]. B lymphocytes differentiate into memory or plasma cells that secrete IgM, IgG, IgE, and IgA isotypes depending on the cellular and cytokine microenvironments [41]. 

B cells initially express only IgM, and isotype switching is T cell-dependent, requiring physical contact in the unique germinal center. The ordered mechanisms of isotype switching are somatic hypermutation (SHM) and class switch recombination (CSR). Single-nucleotide substitutions are initially introduced into the variable region of Ig genes, resulting in IgGs with higher affinity for antigens in a possible second encounter [42]. CSR involves intrachromosomal deletion and recombination. This results in IgG, IgE, and IgA expression by replacement of the heavy-chain constant region (CH) from Cμ to downstream CH segments without affecting their antigen specificity [43]. 

Both SHM and CSR require activation-induced cytidine deaminase (AID), the expression of which is restricted to the germinal center and activated B cells. Nevertheless, the mechanisms underlying SHM and CSR induction remain unclear [44]. B cell-specific AID interacts with and is activated by PKA. Furthermore, pharmacological inhibition of PKA prevents CSR in CH12F3 cells (B cell lymphoma), whereas constitutive PKA activity enhances CSR in mice [45]. This finding suggests that PKA participates in the later stages of B cell differentiation; nonetheless, the mechanism of regulation of this process needs to be investigated.

We discuss the roles of PKA in lymphocyte biology with examples of two immune disorders where T and B cells are affected: CVID and SLE.

## 4. PKA Involvement in Immunological Diseases

### 4.1. PKA in CVID

Primary CVID presents as a heterogeneous phenotype affecting B cell functions such as differentiation, immunoglobulin secretion, survival, and proliferation [9]. However, T cell abnormalities, such as reduced proliferation and regulatory T cell numbers [46], have frequently been found in global cohorts, suggesting impaired T and B cell cooperation. 

Cluster of differentiation 3 (CD3) is expressed on the surface of T cells, where it interacts non-covalently with TCRs that recognize peptides derived from protein antigens coupled to the major histocompatibility complex (MHC) in antigen-presenting cells (APCs). T cells activated using monoclonal anti-CD3 antibodies result in activated downstream TCR signaling that leads to proliferation and cytokine production [47]. The functions of PKA in T cells in patients with CVID have been described based on the suppressive effect of cAMP on T cell proliferation after anti-CD3 stimulation that depends on PKAI [4] formed by the subunits RIα2 and Cβ2. Furthermore, endogenous cAMP levels are higher in T cells derived from patients with CVID with a functional T cell deficiency than in healthy donors. The specific PKAI antagonist Rp-8-bromo-cAMP-phosphorothioate (Rp-8-Br-cAMPS) improves T cell proliferation and IL-2 production, triggered by anti-CD3 stimulation, reaching levels comparable to those in healthy donors [4]. Other findings support the role of PKAI in the inhibition of TCR signaling because tyrosine phosphorylation and *IL-2* mRNA expression are reduced upon CD3/CD28 stimulation in the presence of the cAMP analog 8-(-4-chlorophenylthio)-cAMP (8-CPT-cAMP). The colocalization of RIα with CD3 during capping is associated with the polar mobilization of surface molecules such as TCR, CD3, and CD4/CD8 in T cells, followed by their endocytosis [8]. This supports the notion that PKAI functions in TCR signaling regulation. 

Cytokines such as IL-10 are reduced in CVID, and PKAI is involved in this process [5]. The secretion of IL-10 by T cells after CD3/CD28 activation is reduced in patients with CVID compared with healthy donors, and is reduced in both by 8-CPT-cAMP. However, this effect was more evident in samples from patients. When PKAI was inhibited by Rp-8-Br-cAMPS, IL-10 levels were substantially increased in CD3+ T cells derived from patients and healthy donors. IL-10 is an important anti-inflammatory cytokine, the impaired expression of which correlates with autoimmune processes such as inflammatory bowel disease, resulting in immunopathology and tissue damage [48]. The frequent detection of autoimmune disorders in patients with CVID [49] indicates that PKAI participation could be essential for unbalanced immune responses. Additionally, T regulatory cells (Tregs) are an essential source of I-10 secretion, and they are less abundant in some patients with CVID [50,51].

IL-10 receptors (IL-10Rs) are expressed by immune cells such as macrophages, NK, dendritic, and B cells. The functions of IL-10Rs in B cells include suppressing antigen presentation by MHC class II, as well as inducing proliferation and immunoglobulin production. IL-10 also induces anti-apoptotic Bcl-2 protein expression in B cells located in the specialized germinal centers of secondary lymphoid organs, where B cells are activated and proliferate. These findings indicate that IL-10 is essential for B cell survival. IL-10 can also induce isotype switching to IgG1 and IgG3 in human naïve B cells activated by CD40 [52,53]. Moreover, in vitro activation of B cells in patients with CVID using IL-10 and CD40 improves IgG, IgM, and IgA production [54,55]. These data are notable, given the immunosuppressive effect of IL-10 on monocytes and T cells. The diminished levels of IL-10 in patients with CVID that are sensitive to PKAI activity might influence autoimmune disorders and defects in B cells. 

Overall, these findings suggest the importance of cAMP/PKA signaling in the development of autoimmunity in CVID, as diminished IL-10 levels produced by Tregs represent a mechanism that promotes autoimmunity. In contrast, increased cAMP levels in T cells are associated with diminished activation and proliferation via TCR signaling, which might also impact IgG production and B cell survival. 

### 4.2. PKA in SLE

The autoimmune disorder SLE is characterized by B cell hyperactivation and the production of autoantibodies directed against nuclear components, resulting in the loss of immune tolerance and defective cell suppression. Furthermore, patients with elevated numbers of T cells have a predominant Th2 profile that affects B cell activation. Consequently, patients with SLE have clinical manifestations such as inflammation, vasculitis, immune complex deposition, and vasculopathy. However, the pathogenesis of SLE remains elusive, although it is known that both genetic predisposition and environmental factors can lead to its development [56].

The role of PKA signaling in the development of SLE is not entirely clear. Adenosine binds to adenosine receptors, reduces intracellular cAMP concentrations, and suppresses B cell differentiation and T cell activation. Concentrations of cAMP are 50% lower in T cells from patients with SLE than in those from healthy individuals. However, the intracellular cAMP concentration induced by N6,O2’-di-butyryl adenosine 3’,5’-cyclic monophosphate, a permeable plasma membrane cAMP analog that increases cytoplasmic cAMP levels, is comparable between SLE- and healthy donor-derived T cells. These results suggest that defective adenylate cyclase activity in SLE T cells could influence the autoimmune phenotype [6].

Phosphorylation levels of proteins with R-R-X-S/T motifs (present in PKA substrates) are reduced in T cells from patients with SLE compared with healthy donors. However, phosphorylation is not defective in patients with SLE progression controlled by immunosuppression. This indicates that PKA is involved in exacerbating autoimmunity. Moreover, corticosteroid and/or immunosuppressive agents are not associated with defective phosphorylation of PKA targets in SLE, whether controlled or not [7]. Additionally, capping is diminished in T cells stimulated with CD3 and derived from patients with active SLE [57]. 

Activation of PKAI in T cells is defective in patients with active and mild SLE, as a cAMP agonist interferes with the release of the catalytic subunits [58,59]. Particularly, adenosine does not increase cAMP levels by preventing cAMP from binding to the regulatory subunit of PKAI in T cells from patients with SLE [59]. This indicates that a defect in AC affects T cell activation. Differences between patients with active and controlled SLE indicate that the exact mechanism through which PKAI signaling influences T cell activation in patients with SLE warrants further investigation. 

Similar to CVID, findings in SLE suggest that cAMP signaling could be a target for treatment. Functional defects in PKA are associated with T cell activation in both diseases. However, little is known regarding the effects of PKA on B cells. Additionally, apart from the PKA holoenzyme, AKAPs are crucial for this signaling pathway. The functions of AKAPS in immune cells are described below.

## 5. AKAPs Are Involved in Immunological Processes

AKAPs represent a group of multifunctional, multi-domain proteins that are not structurally related. They are responsible for anchoring signaling molecules that contribute to the sequestering of PKA along with proteins into discrete signalling complexes in specific subcellular enviroments, and thus comprise a critical regulatory mechanism for PKA activity [60]. They share common properties, including a PKA-anchoring domain, organelle localization domains, and the ability to form complexes with other signaling molecules. Moreover, AKAPs contain domains that are important for binding to other signaling proteins, including phosphodiesterases and phosphatases [61], which contribute to accurate regulation.

AKAPs bind to the regulatory subunits via an amphipathic helix of 14–18 residues inserted through the hydrophobic side into the hydrophobic pocket formed by the two regulatory subunits; this promotes localization to several subcellular regions where PKA is required [62]. This is achieved through the capacity of AKAPs to associate with diverse structures such as vesicles, dendrites, the nucleus and membrane, centrosomes, mitochondria, and the endoplasmic reticulum [3].

Most AKAPs bind to RII, whereas some interact with RI, and others anchor to RI and RII subunits. This might influence the differential cellular location of the RI and RII subunits, because PKA types I and II tend to be distributed in the cytosol and subcellular compartments, respectively [63]. Pharmacological approaches, such as using Ht31 peptide derived from AKAP with a high affinity to PKA-R subunits, have facilitated studies of mechanisms through which AKAPs function inside cells and regulate cellular processes. 

The focus of this review is the immune system; therefore, we describe the current findings concerning the role of AKAPs in immune cells. Table 1 summarizes the AKAPs identified in various immune cells and their known functions.

Ezrin, radixin, and moesin comprise the ERM family of proteins that interact with the plasma membrane and actin cytoskeleton. Ezrin is essential for immune responses, as it is associated with membrane rafts formed by the activation of B (BCR) and TCR immunoreceptors. Most immune functions of ezrin are associated with responses to antigen receptors. 

Ezrin colocalizes in the region of an immune synapse between T cells and APCs [64]. Furthermore, ezrin, along with AKAP79 and AKAP149, is found in lipid rafts from dendritic cells, suggesting their participation in antigen presentation [65].

Ezrin is also associated with the migration of various cell types. Specifically, it interacts with Myo18aα in B cells [66], which regulates cell migration [67]. Therefore, ezrin might be important for B cell migration in response to chemokines.

Finally, B cell development is unaltered in murine models of a conditional ezrin deficiency in B cells, whereas the response to BCR activation leads to increased proliferation and differentiation into antibody-secreting cells [68].

AKAPs are also important for macrophage function. Silencing AKAP10 and AKAP11 diminishes nitric oxide (NO) synthesis, and NO inducers such as LPS or prostaglandin (PGE)-2 do not restore NO levels in RAW264.7 murine macrophage cells. Furthermore, using the RIAD peptide to disrupt AKAP binding to RI subunits leads to diminished IL-10 and TNF-α levels even in alveolar macrophages activated by LPS exposure [69].

In conclusion, PKA seems to be important in the development of autoimmune diseases such as SLE. The expression of AKAP9 and AKAP79 is elevated in T cells from patients with SLE; nevertheless, the causes remain unknown [70].

**Table 1:** Main A-kinase anchoring proteins (AKAPS) that participate in immune cell functions.

Name	Cell Types	Functions and References
Ezrin	T, dendritic, and B cells	Activation by immunoreceptors [64], antigen presentation [65], migration [68], and B cell proliferation and differentiation [71].
AKAP149	Dendritic cells	Antigen presentation [65].
AKAP79	Dendritic cells	Antigen presentation [65]. Elevated in T cells of patients with SLE [70].
AKAP10	Alveolar macrophages	LPS-induced NO synthesis, IL-10 and IL-6 synthesis mediated by PGE [69].
AKAP11	Alveolar macrophages	PGE suppression of TNF-α [69].
AKAP450	T cells	T cell motility induced via LFA-1 integrins [72].
AKAP9	T cells	Elevated in T cells of patients with SLE [70].
LRBA	B cells	Physical interaction reported [11].

AKAPs are abundant in T cells and APCs, whereas only the AKAPs ezrin and LRBA are known to function in B cells. Mutations involving *LRBA* are associated with CVID [51], and although LRBA protein is expressed in immune cells, its role in B cells has not been elucidated. The findings in lymphocytes are discussed below. 

LRBA protein is a ubiquitous cytosolic high-MW protein (larger isoform, 319 kDa) with inducible expression in immune cells such as lymphocytes and macrophages [10]. Mutations in *LRBA* have been associated with CVID. A deficiency in LRBA is characterized by low B cell counts, defective differentiation, hypogammaglobulinemia, autoimmunity, and lymphoproliferative disorders [51]. A fully tertiary LRBA structure has not yet been elucidated, and it is found only in pleckstrin-homology (PH) beige and Chediak–Higashi (BEACH) domains [73]. However, based on homology with other proteins, some of its predicted domains might be scaffolding proteins [74].

The expression of LRBA in humans and mice is inducible by LPS, and homology analysis has revealed an ortholog, DAKAP550, in *Drosophila melanogaster* that acts as an AKAP. The secondary structure of amphipathic helix on LRBA has been determined based on the knowledge of AKAP binding sites and PKA regulatory subunits [10]. Physical interactions between LRBA and the RIIβ regulatory subunit have recently been identified in human B cells using co-immunoprecipitation assays in Ramos B lymphoma cells. This interaction with RIIα and RIIβ has also been identified in human primary B cells stimulated with CD40L and IL-4 [11]. Additionally, LRBA interacts with RIIβ subunits in kidney tissues, and these interactions are important for aquaporin-2 phosphorylation by PKA [75]. These findings support the notion that LRBA functions as an AKAP.

AKAPs also interact with cellular organelles and PKA substrates, and LRBA contains predicted domains that might interact with various cellular structures and proteins that are characteristic of AKAPs. Towards its N-terminus, LRBA has a concanavalin (Con)-A-like domain and a VHS domain [VPS (vacuolar protein sorting)-27, Hrs (hepatocyte growth factor-regulated tyrosine kinase substrate)] that are related to interactions with specific cellular structures such as vesicles [10]. Immunoprecipitation assays have shown that interactions between LRBA and the intracellular domain of CTLA-4 in endocytic vesicles prevent CTLA-4 binding to the lysosomal protein AP-1, thus preventing CTLA-4 degradation and recycling [76]. The domains PH and BEACH are important for vesicular trafficking and essential for protein folding, as the crystal structures of these domains have revealed intramolecular interactions [73].

A sequence of WD40 repeats located toward the carboxy terminal of LRBA can bind to molecules with different patterns and might be important in vesicular trafficking as well as autophagy. The site is a predicted LC3-interacting region (LIR) for that part of the protein [74]. Additionally, WD40 might be important for the generation of LRBA homo- and heterodimers. Although these domains predict the function of LRBA, they are consistent with other characteristics of AKAPs in that they also have additional domains that allow interactions with various structures and molecules.

**Figure 1:**
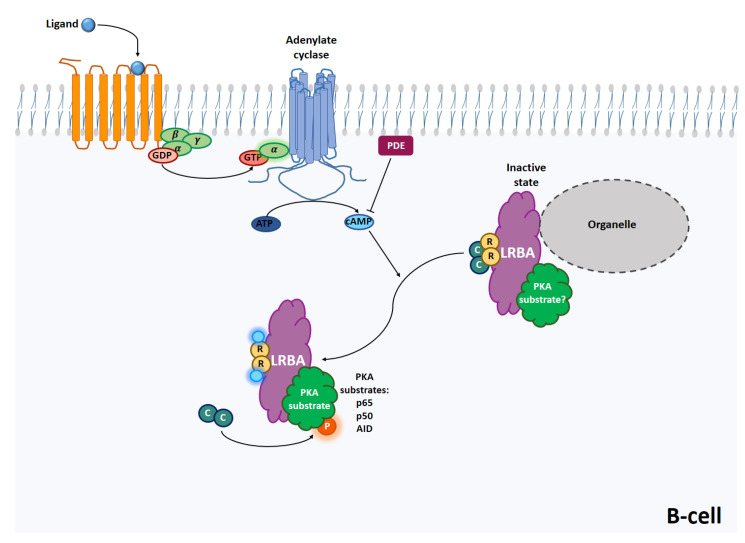
Activation of PKA requires interaction of GPCRs with hormone and chemokine ligands. Such interactions activate G-proteins to generate guanine-exchange factor activity, where Gα dissociates from β and γ and induces production of GTP that activates ACs that, in turn, induce the production of cAMP from ATP. Cyclic AMP binds to regulatory subunits of PKA that activate catalytic subunits to phosphorylate PKA protein targets in B cells such as p50, p65, and AID. AKAPs such as LRBA participate in this signaling by localizing PKA in specific subcellular sites and by interacting with PKA targets. This signaling is regulated by the activation of phosphodiesterases, ACs (adenylyl cyclases), GPCRs (G-protein coupled receptors), LRBA protein, PKA, and PLA.

The elucidation of PKA targets regulated by interactions with LRBA could explain the reduced proliferation of B cells, isotype switching, abrogated immunoglobulin production in B cells isolated from patients with LRBA deficiency [51], hypogammaglobulinemia, and even autoimmunity (Figure 1).

## 6. Conclusions

The number of investigations into PKA signaling in immune cells is increasing. The identification of new AKAPs expressed under activation by proteins such as LRBA will provide insights into immune regulation and facilitate understanding of immunological diseases. 

## Data Availability

Not applicable.
